# Supplementary material for: Rational design and dynamics of self-propelled colloidal bead chains: from rotators to flagella
Source: Sci Rep. 2017 Dec 1;7:16758. doi: 10.1038/s41598-017-16731-5 (PMC5711812; doi:10.1038/s41598-017-16731-5)
Supplement: Supplementary file 10 — Supplementary Information [file 41598_2017_16731_MOESM10_ESM.pdf]

Supplementary Information

Rational design and dynamics of self-propelled colloidal bead chains: from rotators to flagella

Hanumantha Rao Vutukuri[a,*,||], Bram Bet[b], René van Roij[b], Marjolein Dijkstra[c,*], Wilhelm T. S. Huck[a,*]

[a]Institute for Molecules and Materials, [a]Radboud University, Heyendaalseweg 135, 6525 AJ Nijmegen, The Netherlands

[b]Institute for Theoretical Physics, Center for Extreme Matter and Emergent Phenomena, Utrecht University, Leuvenlaan 4, 8584 CE Utrecht, The Netherlands

[c]Soft condensed Matter, Debye Institute for Nanomaterials Science, Utrecht University, 3584 CH, The Netherlands

[||] Present address: Soft Materials, Department of Materials, ETH Zurich, 8093 Zurich, Switzerland

Email: hanumantharaov@gmail.com, m.dijkstra@uu.nl, & W.Huck@science.ru.nl

Supplementary Movies:

Movie S1: This movie shows the active rotational motion of a stiff 6-bead chain in 1.0 *vol*% H_2O_2. The movie was acquired at 20 fps and it is played at 40 fps.

Movie S2: This movie shows the artificial flagella-like motion when a semiflexible 6-bead chain is transferred to 1.0 *vol*% H_2O_2. The movie was acquired at 20 fps and it is played at 40 fps.

Movie S3: This movie shows the propelling behavior of a half-stiff and half-semiflexible 8-bead chain in 1.0 *vol*% H_2O_2. The movie was acquired at 10 fps and it is played at 20 fps.

Movie S4/animation 1: This animation shows the motion of a zig-zag bead chain, with a 'zig-zag' arrangement of effective propulsion forces.

Movie S5/animation 2: This animation shows the motion of a C-shaped bead chain, corresponding to shape parameters $r/\sigma = 0.25$ and $p/\sigma = 0$, with a 'zig-zag' arrangement of effective propulsion forces.

Movie S6/animation 3: This animation shows the motion of a helically shaped bead chain, corresponding to shape parameters r/σ = 0.88 and p/σ = 6.2, with a 'zig-zag' arrangement of effective propulsion forces.

Movie S7/animation 4: This animation shows the motion of a zig-zag bead chain, with a heterogeneous distribution of effective propulsion forces.

Movie S8/animation 5: This animation shows the motion of a C-shaped bead chain, corresponding to shape parameters r/σ = 0.25 and p/σ = 0, with a heterogeneous distribution of effective propulsion forces.

Movie S9/animation 6: This animation shows the motion of a helically shaped bead chain, corresponding to shape parameters r/σ = 0.88 and p/σ = 6.2, with a heterogeneous distribution of effective propulsion forces.

Supplementary Figures

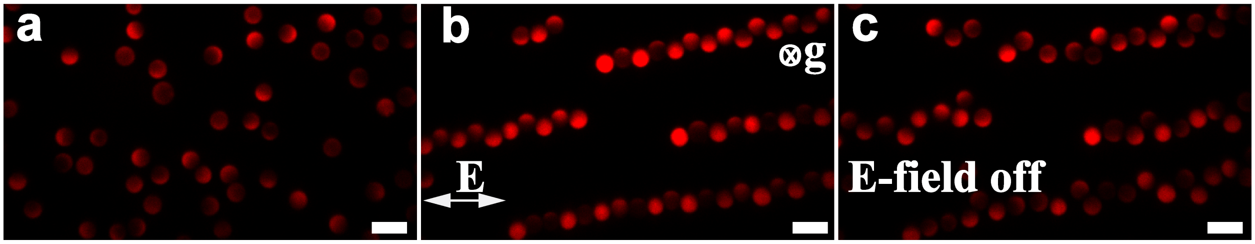

SFigure 1. Confocal micrographs show the response of the Janus particles in AC electric fields. (a) In the absence of the field. (b) In the presence of the field (E_{rms} = 0.01 Vμm^{-1}, f = 800 kHz) particles assembled into staggered or zig-zag chains. The dark side of the particle represents the Pt coated side. The arrow indicates the direction of the applied electric field. (c) Shortly after switching off the field. Scale bars are 2.0 μm.

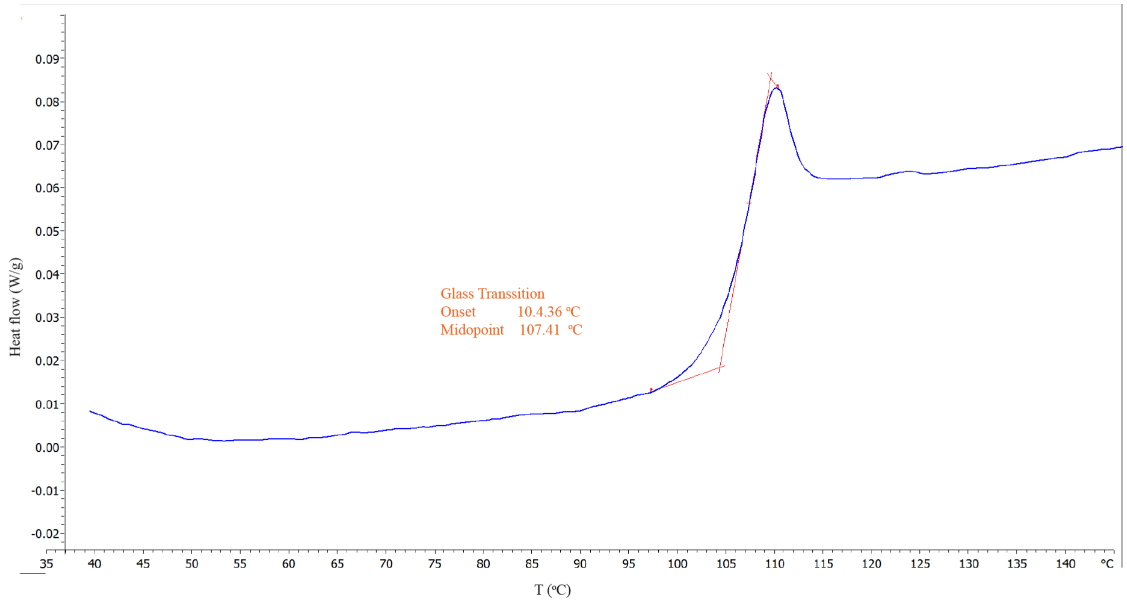

SFigure 2. Differential scanning calorimetric diagram of 1.35 μm sized sterically stabilized PS particles.

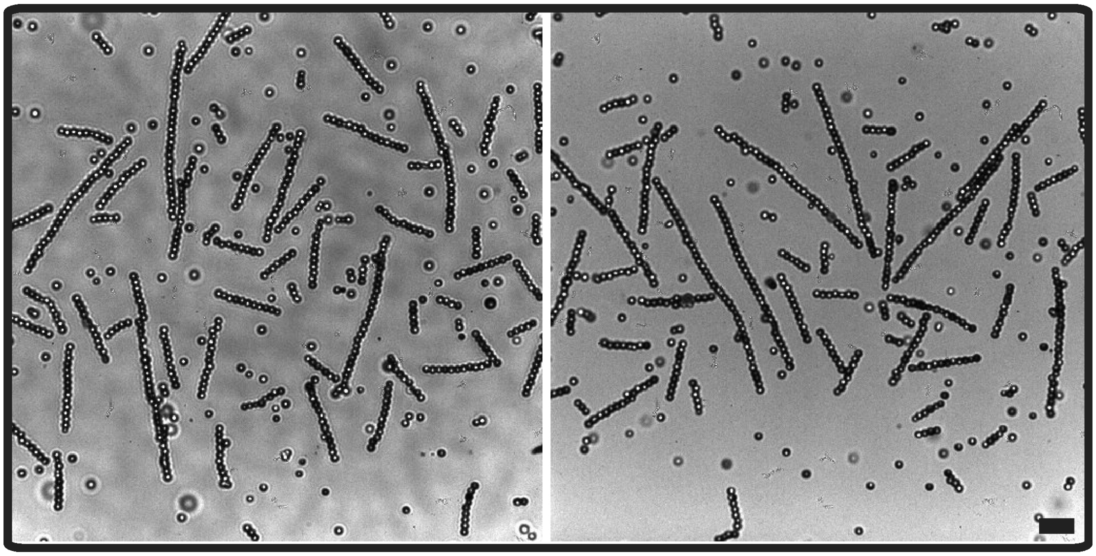

SFigure 3. Bright field optical micrographs of stiff active bead chains at different locations in the sample cell. Scale bar is 10.0 μm.

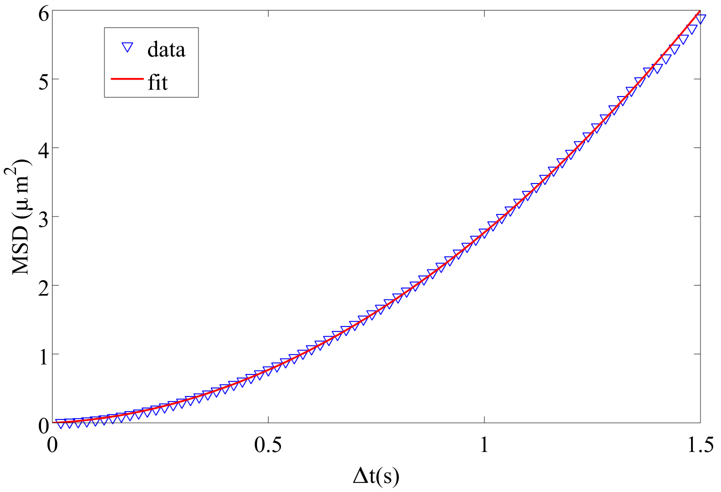

SFigure 4. Mean-squared displacement (MSD) of self-propelled singlet spheres in 1.0 vol% H_2O_2 . The solid red line is the fit from the MSD equation ($\langle \Delta r^2 \rangle = \langle [r(t) - r(0)]^2 \rangle = v^2 t^2 + 4 D_t t$).

Bead-shell model

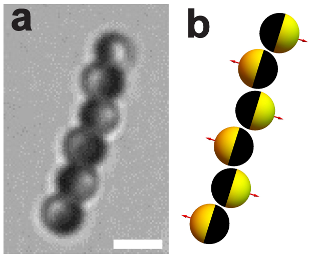

S**Figure 5**. Bright field micrograph (a) of a rigid bead chain composed of 6 Janus spheres. From the different colors, a 'zig-zag' arrangement can be clearly observed (a). Illustration (b) of a similar bead chain. The arrows indicate the direction of the effective propulsion force. Scale bar is 5. 0 μm.

Rigid bead chains		
Length bead chain (σ)	ω_{exp} (rad/s)	ω_{calc} (rad/s)
2	2.61± 0.20	2.75
4	1.49± 0.13	1.16
6	0.48± 0.08	0.65

Table SI: A comparison between the experimentally observed angular velocity and the angular velocity as calculated from the average effective propulsion force using the resistance tensor, for bead chains of different length.

Rigid bead chains		
Number of beads in a chain	Active (1.0 vol% H_2O_2) ω (rad/s)	Passive D_r (s^{-1})
2	2.61 ± 0.2	0.092 ± 0.03
3	1.2 ± 0.25	0.041 ± 0.02
4	1.49 ± 0.13	0.023 ± 0.013
5	0.9 ± 0.27	0.014 ± 0.016
6	0.48 ± 0.08	0.005 ± 0.002

(semi-)flexible bead chains		
Number of beads in a chain	Active (1.0 vol% H_2O_2) ω (rad/s)	Active (1.0 vol% H_2O_2) v (μm/s)
6	3.5± 0.5	0.9± 0.1
8	3.8± 0.9	0.62 ± 0.12

Table SII: Experimentally measured rotational speed and diffusivity of rigid bead chains. Rotational and translational speed of semi-flexible bead chains.

Semi-flexible bead chains

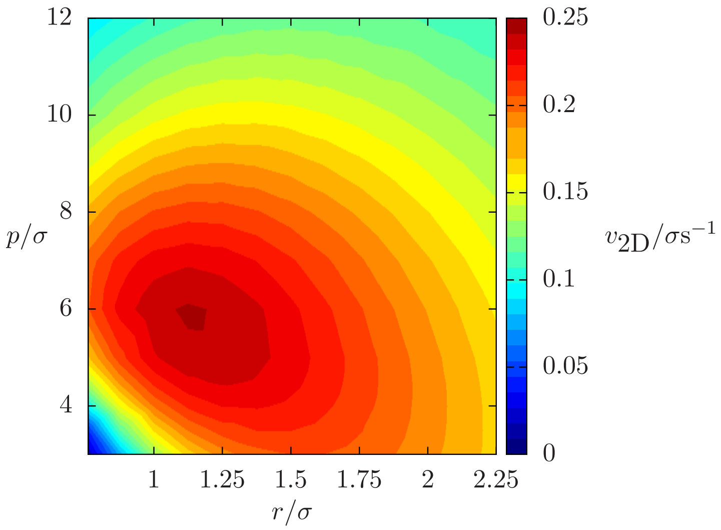

SFigure 6. Contour plot of the in-plane (projected) velocity v_{2D} in units of the diameter of the Janus particle σ per second, as a function of helical radius r/σ and pitch p/σ, for a typical heterogeneous force distribution. The color bar denotes the magnitude of v_{2D}.

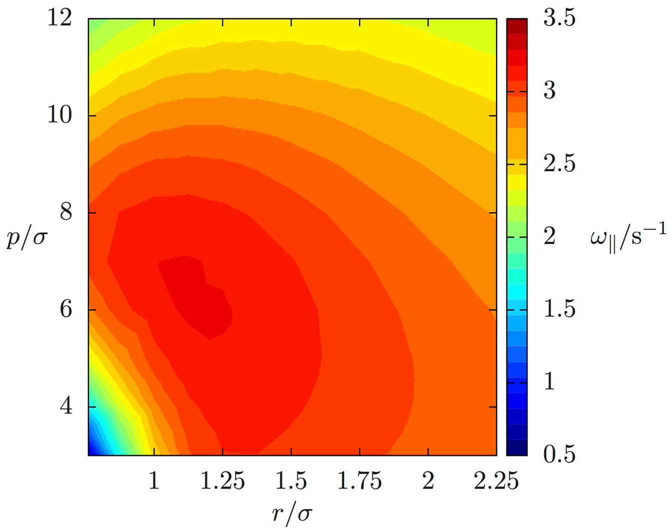

SFigure 7. Contour plot of the angular velocity $\omega_{\|}$ around the body axis (connecting the end beads), as a function of helical radius r/σ and pitch p/σ, for a typical heterogeneous force distribution. The color bar denotes the magnitude of $\omega_{\|}$.
